# Manufacturing of Bioinspired SS316L-Based Multimaterials: Processing, Mechanical Properties and Modeling

**DOI:** 10.3390/mi17060699

**Published:** 2026-06-08

**Authors:** Vinod Kumar Darapureddy, Tuhin Mukherjee, Sonia Mary Chacko, Zahabul Islam

**Affiliations:** 1 Mechanical and Manufacturing Engineering, School of Engineering, Bowling Green State University, Bowling Green, OH 43403, USA; 2 Mechanical Engineering, Iowa State University, Ames, IA 50011, USA; 3 Robotics Engineering, School of Engineering, Bowling Green State University, Bowling Green, OH 43403, USA

**Keywords:** additive manufacturing, laser powder bed fusion (LPBF), SS316L-Cu multimaterial, bioinspired lattice structures, molecular dynamics (MD) simulation

## Abstract

This study presents a hybrid additive manufacturing approach to fabricate bioinspired stainless steel 316L-copper (SS316L-Cu) multimaterial structures using laser powder bed fusion (LPBF). The present study incorporates honeycomb lattice structures with varying wall thicknesses (0.25 mm, 0.5 mm, 0.75 mm, and 1.0 mm) to investigate the effect of geometric parameters on mechanical performance. Mechanical testing was conducted according to ISO 6892 standards, and the results revealed a strong dependence of tensile strength and ductility on lattice thickness. Copper (Cu) infiltration into SS316L lattice structures improved ductility by 30% compared to the monolithic SS316L lattice, with minimal compromise in tensile strength. To complement experimental results, molecular dynamics (MD) simulations were performed to study atomic-scale deformation and validate the trend of strength enhancement with increasing wall thickness. The findings demonstrate the potential of combining LPBF and liquid Cu infiltration to develop multifunctional, mechanically robust, and thermally conductive metallic composites. This approach provides valuable insight into structure–property relationships and supports the design of next-generation multifunctional composites for structural and thermal applications.

## 1. Introduction

The advancement of additive manufacturing has opened new pathways in the design and fabrication of functionally graded, structurally optimized, and performance-tailored components [1,2,3,4,5,6,7,8,9,10,11,12,13,14,15]. Multimaterials refer to the integration of two or more distinct materials within a single structure to achieve a combination of mechanical, thermal, or electrical properties that cannot be attained by monolithic materials alone [16,17,18,19]. Among various metal-based combinations, stainless steel 316L and copper (SS316L-Cu) multimaterials have attracted considerable interest due to their synergistic potential, combining the high strength, corrosion resistance, and biocompatibility of SS316L with the excellent thermal and electrical conductivity of copper [20,21,22,23]. These properties make SS316L-Cu multimaterials particularly attractive for applications in heat exchangers, mold tooling, biomedical implants, and structural-electronic integration.

Recent research in metal additive manufacturing [1,3,4,12,24,25,26,27,28,29,30,31] and hybrid additive manufacturing [32,33,34,35] has explored novel strategies for the fabrication of multimaterial metal systems. LPBF offers unparalleled geometric precision and design freedom for complex lattice or cellular structures, such as bioinspired honeycombs and triply periodic minimal surfaces (TPMS), to name a few [15,31,36,37,38,39,40,41,42,43]. However, direct LPBF processing of metallurgically incompatible metal pairs remains challenging due to issues such as poor interfacial bonding, cracking, delamination, and significant differences in thermal expansion and melting points [44]. To overcome these challenges, several hybrid approaches have emerged, including sequential printing, in situ alloying, and post-processing infiltration techniques. In the context of SS316L-Cu systems, copper infiltration into SS316L lattice structures might be effective in mitigating interfacial defects while enabling dense, continuous copper networks that enhance thermal performance. Despite its potential, there remain gaps in understanding how the geometrical [42] features of lattice structures, such as lattice thickness (t) and unit cell size (u), influence infiltration efficiency, interfacial bonding, and overall mechanical behavior of the final composite.

In this study, we present a hybrid manufacturing approach that combines LPBF and post-process copper infiltration to fabricate bioinspired honeycomb SS316L-Cu multimaterial structures. Bioinspired honeycomb architectures were selected in the present study because they provide a well-defined cellular framework for systematically evaluating the influence of wall thickness and relative density on mechanical performance. Honeycomb structures are widely used in lightweight load-bearing, impact-resistant, and energy-absorbing applications because their wall-dominated deformation behavior enables clear interpretation of structure–property relationships. Although three-dimensional architectures such as TPMS and other lattice structures may offer different mechanical behavior and interconnected pathways, the honeycomb design used in this study allowed direct investigation of wall-thickness-dependent deformation and enabled examination of the in-plane SS316L–Cu interfacial region and its role in load transfer. SS316L lattice structures were first printed using LPBF with varying lattice thicknesses (t = 0.25 mm, 0.5 mm, 0.75 mm, and 1 mm) and unit cell sizes (u_x_ = 2 mm and u_y_ = 3 mm in the x and y directions, respectively). Following manufacturing, a copper infiltration technique was employed to fill the voids, producing dense SS316L-Cu composites. Tensile testing was conducted on both the as-printed SS316L lattice structures and the infiltrated SS316L-Cu multimaterial samples to evaluate the mechanical performance enhancement imparted by copper incorporation. This study aims to provide insights into the structure–property relationships of multimaterial architectures to demonstrate a scalable pathway for fabricating advanced SS316L-Cu composites using hybrid manufacturing strategies.

## 2. Materials and Methods

### 2.1. Design of Bioinspired Honeycomb Structures

**Figure 1:**
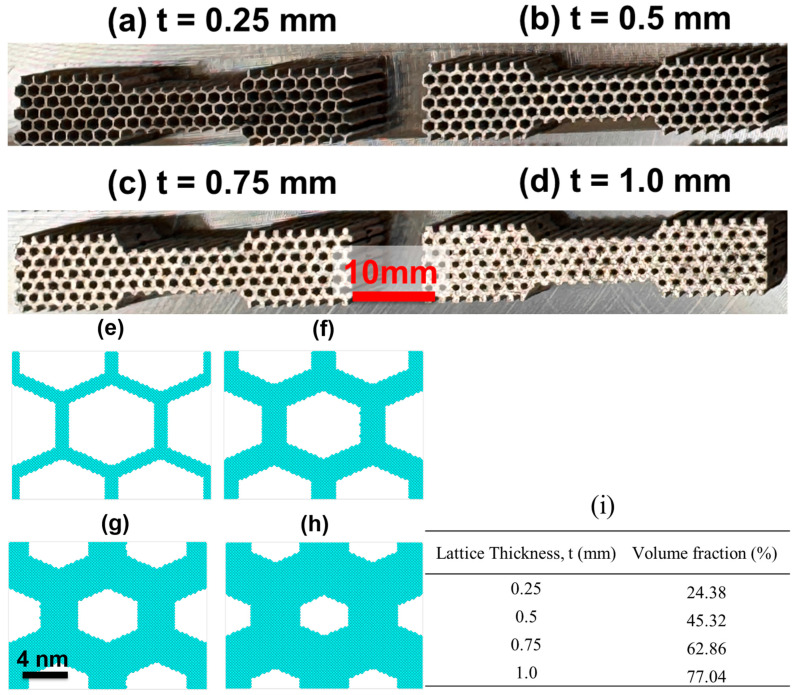
(**a**) Honeycomb design of hexagonal lattice with varying lattice wall thickness: (**a**–**d**) LPBF printed lattice, computer model for md simulation with volume fraction: (**e**) 24.38%, (**f**) 45.32%, (**g**) 62.86%, (**h**) 77.04% and (**i**) lattice thickness and corresponding volume fraction of additive manufactured SS316L.

Bioinspired honeycomb structures were designed to serve as lattice structures for SS316L-Cu multimaterial composites. The four different Lattice wall thicknesses of 0.25 mm, 0.5 mm, 0.75 mm, and 1 mm were selected for this study (Figure 1a–d). These lattice thickness corresponds to 24.38%, 45.32%, 62.86% and 77.04% volume fraction of SS316L. For all four design unit cell sizes were selected as 2 mm in the x direction and 3 mm in the y direction. These design variations were intended to study the influence of structural geometry on mechanical performance. All lattice designs were created using nTop software (5.21.2) [45] and exported in STL format for further slicing and printing.

### 2.2. Additive Manufacturing via LPBF

The SS316L lattice structures were fabricated using a commercial Laser Powder Bed Fusion (LPBF) system from Xact Metal equipped with a 400 W, 50 µm dual spot fiber laser. Gas-atomized SS316L powder (15–45 µm particle size) was used as the feedstock material for LPBF printing. The quality of LPBF-fabricated components depends on the combined influence of process parameters including laser power (P), scan speed (v), hatch spacing (h), and layer thickness (t), which collectively govern melt pool characteristics, thermal history, and densification behavior. Variations in these parameters can significantly influence porosity, fusion quality, microstructure, and mechanical properties of printed components. In the present study, manufacturer-recommended optimized LPBF process parameters consisting of a laser power of 180 W, scan speed of 1900 mm/s, hatch spacing of 50 µm, and layer thickness of 30 µm were employed to ensure stable processing and fabrication of high-density specimens (relative density~99.5%). The selected parameter combination was therefore considered suitable for achieving sufficient melt pool stability, inter-track bonding, layer consolidation, and overall fabrication quality for the SS316L lattice structures.

All samples were printed in an Argon-purged chamber to minimize oxidation (<0.2% Oxygen) during the printing.

Following LPBF, the porous SS316L lattice structures were subjected to a copper infiltration process to fabricate SS316L-Cu multimaterials. Following LPBF fabrication, the high-density (relative density~99.5%) SS316L lattice structures were converted into SS316L–Cu multimaterial structures using a mold-assisted Cu (high purity 99.9%) infiltration process. The detailed Cu infiltration procedure, including Cu purity, melting temperature, feeding method, vacuum assistance, and cooling conditions, is provided in Supplementary Section S1.

### 2.3. Mechanical Testing

Uniaxial tensile tests were conducted on both as-printed SS316L lattice samples and copper-infiltrated SS316L-Cu multimaterial composites to evaluate the effect of Cu infiltration on mechanical performance. All tensile tests were performed using a universal testing machine (e.g., Shimadzu 50 kN) following ISO 6892 standards [46]. A constant strain rate of 2 mm/min was applied during the tensile testing. The ultimate tensile strength, σ_u_ (UTS), yield strength (σ_Y_), Young’s modulus (E), and failure strain (ε_f_) were recorded for all samples.

### 2.4. Molecular Dynamics Simulation Study

The corresponding atomistic models of honeycomb are shown in Figure 1e–h. In this study, a time step of 1 femtosecond (fs) and a simulation pressure of 1 bar were maintained throughout all simulations. Both body-centered cubic (BCC) iron and face-centered cubic (FCC) Cu simulation cells had approximate dimensions of 200 Å × 1500 Å × 50 Å. The lattice constants for the BCC unit cell of Fe and the FCC unit cell of Cu were set to 2.866 Å and 3.6149 Å, respectively. At the beginning of each simulation, all structures were relaxed to an energy-minimized state using the conjugate gradient (CG) method. Periodic boundary conditions were applied in all three spatial directions. Following energy minimization, NPT (constant number of particles, pressure, and temperature) dynamics were performed for several thousand steps to further relax the atomistic models. All simulations were conducted using the LAMMPS simulation package [47]. Embedded Atom Method (EAM) potential [48] is employed to study the lattice structure using the LAMMPS code [47]. The EAM potential is used to define the total energy of the system and is defined by the following expressions:

(1) $$U=\sum_{i}F\left(\rho_{i}\right)+\frac{1}{2}\sum_{i}\sum_{i\neq j}\varphi_{ij}$$

where U represents the total energy of the system, $F\left(\rho_{i}\right)$ is the embedded energy as a function of the electron density, $\rho_{i}$ denotes the electron density contributed by the host atoms, and $\varphi_{ij}$ is the pairwise interaction dependent on the distance between atoms i and j. During this simulation, a strain rate of 0.001 Å/ps was then selected, and NPT dynamics were employed for time integration during the tensile loading.

## 3. Results and Discussion

### 3.1. Tensile Performance of SS316L Lattice Structures

**Figure 2:**
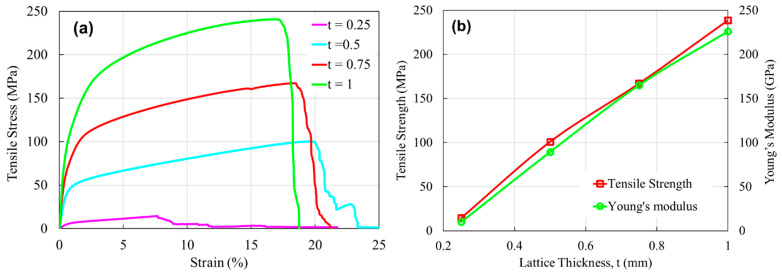
(**a**) Tensile response of lattice structures with different lattice wall thicknesses (t in mm) and (**b**) variation in tensile strength and Young’s modulus with lattice thickness.

The tensile stress–strain response of laser powder bed fusion (LPBF)-fabricated SS316L lattice structures with varying wall thicknesses (t = 0.25 mm, 0.50 mm, 0.75 mm, and 1.00 mm) is shown in Figure 2a. All stress–strain curves presented in this study represent engineering stress–strain behavior. The results demonstrate a strong dependency of tensile performance on the lattice thickness, t. Thinner lattice walls (t = 0.25 mm) showed low tensile strength (~15 MPa) and early fracture at lower strains, whereas increasing the wall thickness to 1.00 mm significantly enhanced the ultimate tensile strength (~240 MPa) and failure strain (~21%), indicating improved load-bearing capacity and ductility. Thicker cell walls (or increased lattice thickness) directly lead to greater cross-sectional area, enabling the structure to carry more tensile load before failure.

The stress–strain curves reveal typical characteristics of additively manufactured SS316L, including an initial linear elastic region followed by gradual yielding and strain hardening. Samples with thicker walls demonstrate high strength, suggesting larger cross-sectional area enables the structure to carry more tensile load before failure. These findings confirm that lattice thickness is a critical parameter in determining the mechanical response of LPBF-fabricated SS316L lattice structures. Increasing thickness enhances load-bearing capability and postpones the onset of failure, making thicker structures more suitable for applications demanding higher structural integrity. However, these benefits must be balanced against potential trade-offs such as increased material usage and weight, which may affect design decisions for lightweight or energy-absorbing components.

### 3.2. Effect of Lattice Thickness on Tensile Strength and Young’s Modulus

**Figure 3:**
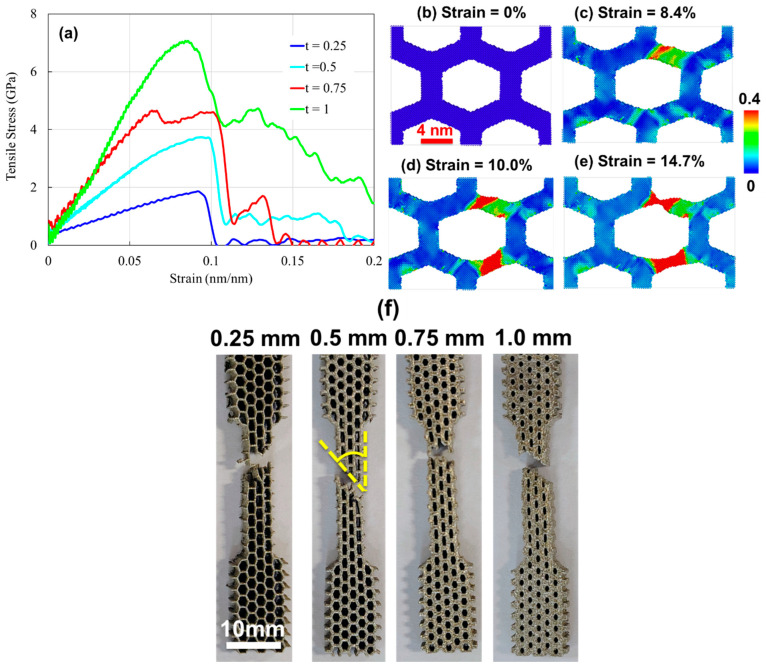
MD simulation study: (**a**) tensile response of lattice structures with different lattice wall thickness, (**b**–**e**) MD simulation snapshots showing shear strain distribution at different applied strain levels, and (**f**) samples after tensile failure.

The variation in tensile strength as a function of lattice wall thickness (t) for SS316L lattice structures fabricated using LPBF is shown in Figure 2b. For thin lattice structures, minor differences between the designed and manufactured dimensions can arise due to melt pool behavior, partially fused powder particles, and surface roughness effects associated with LPBF processing. These effects are commonly observed in LPBF-manufactured thin features and may influence the effective cross-sectional area. However, the optical observations of the representative lattice structure with t = 0.75 mm, shown in Figure 3, confirm good agreement between the designed and fabricated geometry. This indicates that the optimized LPBF parameters provided satisfactory dimensional accuracy, consistent feature formation, and repeatable manufacturing quality for the SS316L lattice structures. The data indicate a strong positive correlation between increasing wall thickness (t) and the tensile strength of the printed lattices. At the smallest lattice thickness (t = 0.25 mm), the tensile strength was relatively low (~15 MPa), reflecting limited structural robustness and a higher likelihood of premature failure due to thin strut geometry and potential LPBF-induced defects. As the lattice thickness increased to 0.50 mm, the tensile strength rose substantially to ~100 MPa, suggesting enhanced mechanical stability and improved distribution of applied stress throughout the structure. Further increases in wall thickness to 0.75 mm and 1.00 mm led to tensile strengths of ~170 MPa and ~240 MPa, respectively.

These improvements are attributed to greater cross-sectional areas for load transfer, reduced geometric imperfections, and less stress concentrations. Thicker lattice elements offer more resistance to stress concentrations, contributing to overall structural integrity under tensile loading.

Figure 2b also illustrates the dependence of Young’s modulus on lattice thickness (t) for SS316L lattice structures manufactured by LPBF. A clear trend is observed: stiffness increases with increasing lattice wall thickness, underscoring the impact of geometric design on elastic response. The thinnest structure (t = 0.25 mm) exhibited the lowest Young’s modulus (~15 GPa), suggesting limited resistance to elastic deformation. This low stiffness is likely a result of reduced effective cross-sectional area and the higher compliance of thin-walled structures, which are more prone to bending and local distortions under tensile loads. As the lattice thickness increased to 0.50 mm and 0.75 mm, Young’s modulus improved significantly to ~90 GPa and ~165 GPa, respectively. These changes reflect enhanced rigidity due to thicker struts, which provide greater resistance to deformation. The highest modulus (~230 GPa) was recorded for the lattice with t = 1.00 mm, where the structure behaved more like a solid than a compliant lattice due to the high-volume fraction of the SS316L compared to the empty or void area.

These results highlight that lattice thickness or volume fraction of SS316L is a key design parameter in optimizing the mechanical performance of metallic lattice structures produced by additive manufacturing. With careful tuning t, the tensile strength of SS316L lattices can be tuned to meet application-specific requirements in fields such as biomedical implants, aerospace, and structural components. In addition to geometric effects, the mechanical behavior of LPBF-fabricated structures can also be influenced by process-induced porosity and local defects. In this study, manufacturer-recommended optimized LPBF processing parameters as discussed in Section 2.2 were employed to minimize defect formation and ensure consistent fabrication quality. The observed increase in tensile strength and Young’s modulus with increasing lattice wall thickness suggests that structural geometry was the dominant factor governing mechanical behavior.

To further quantify the relationship between the volume fraction of the lattice and mechanical performance, the variation in ultimate tensile strength and Young’s modulus with the effective volume fraction of the lattice structure was analyzed using a power-law formulation and can be correlated through a power-law relationship [49,50]:

(2) $$E=C_{1}{{(\rho }^{*})}^{n}$$

(3) $$\sigma_{u}={{C_{1}(\rho }^{*})}^{n}$$

where E is the elastic modulus, $\sigma_{u}$ is ultimate tensile strength, C_1_ is a material-dependent constant, ρ* represents the relative density, and n is the scaling exponent. The volume fraction of the SS316L lattice structure is defined as follows:

(4) $$\rho^{*}=\frac{V_{hexagonal}}{V_{Bulk}}$$

The coefficients C_1_ and n for the hexagonal lattice structure were determined from power-law fitting. These parameters provide important insight into the deformation mechanisms and can be used as inputs for multiscale simulations. The Young’s modulus of the hexagonal lattice structure in terms of volume fraction can be expressed as:

(5) $$E=0.363{{(\rho }^{*})}^{1.761}$$

In addition to the elastic modulus, the dependence of strength on relative density was also evaluated. The ultimate tensile strength of the hexagonal lattice structure in terms of volume fraction can be written as:

(6) $$\sigma_{u}=0.359{{(\rho }^{*})}^{1.616}$$

The results reveal a strong nonlinear dependence of both strength and stiffness on the volume fraction. The scaling exponent for tensile strength (n ≈ 1.62) indicates that strength increases nonlinearly with increasing relative density. This behavior suggests that, beyond simple cross-sectional area effects, additional mechanisms such as improved load transfer, reduced stress concentration, and enhanced structural stability contribute to strength enhancement. As the lattice transitions from a highly porous structure to a denser framework, the dominant deformation mode shifts from bending-dominated to stretching-dominated behavior, resulting in a more efficient load-bearing response. Similarly, the Young’s modulus exhibits an even higher scaling exponent (n ≈ 1.76), indicating a stronger sensitivity of stiffness to volume fraction. This trend is consistent with classical cellular solid models, where elastic modulus is highly dependent on structural connectivity and strut thickness. At lower volume fractions, deformation is governed by bending of thin cell walls, leading to low stiffness. As the volume fraction increases, thicker struts and improved nodal connectivity suppress bending and promote axial load transfer, significantly enhancing rigidity. Overall, the observed power-law behavior establishes a clear structure–property scaling relationship for LPBF-fabricated SS316L lattice structures. These results are consistent with Gibson–Ashby-type models for cellular solids [51] and confirm that mechanical properties can be predictively tuned through architectural design. The ability to control scaling exponents through geometry provides a powerful pathway for optimizing lattice-based multimaterial systems for targeted applications requiring a balance of strength, stiffness, and weight efficiency.

### 3.3. Tensile Behavior of SS316L Lattice Structures: MD Simulation Study

The tensile stress–strain curves obtained from molecular dynamics (MD) simulations of SS316L lattice structures with lattice wall thicknesses of t = 0.25 mm, 0.50 mm, 0.75 mm, and 1.00 mm are shown in Figure 3a. The simulations were conducted to complement experimental data and explore atomic-scale deformation mechanisms. As expected, the peak stress values predicted by MD are significantly higher than those observed experimentally, due to the difference in length scale, time scale, strain rate, and the absence of defects in the simulated models. It should be noted that the tensile strengths predicted by MD simulations are significantly greater than experimentally measured values because of fundamental differences in length scale, time scale, and loading conditions. The atomistic models assume idealized defect-free, and MD simulations require extremely high strain rates for computational feasibility, often several orders of magnitude larger than experimental strain rates. However, the selected strain rate is in good agreement with previous studies and was chosen to minimize strain-rate effects [42,52,53]. Such conditions suppress time-dependent deformation mechanisms and typically result in higher predicted strengths. Therefore, the MD simulations in this study should primarily be interpreted as a technique for understanding deformation trends and atomic-scale mechanisms rather than for direct quantitative prediction of experimental strength values. Quantitatively, the maximum tensile stress reached in the t = 0.25 mm sample was approximately 1.9 GPa, increasing to 3.6 GPa, 5.0 GPa, and 7.1 GPa for wall thicknesses of 0.5 mm, 0.75 mm, and 1.0 mm, respectively. In contrast, the corresponding experimental strengths for these thicknesses were low. While the magnitudes differ due to idealized simulation conditions, the trend is remarkably consistent: tensile strength increases monotonically with lattice thickness in both MD and experimental results. The MD simulation exhibits a clear elastic regime prior to yielding. After reaching peak stress, all samples exhibit strain softening, likely associated with shear band nucleation and local failure propagation. This trend suggests that thicker lattice struts support more loads before complete failure. These simulations confirm that geometric design, particularly lattice thickness, is a governing factor in dictating the strength and failure mode of architected SS316L structures.

### 3.4. Comparison of Failure Behavior Between Experimental and MD Simulation

A comparison between experimental investigation and MD simulation study of the post-failure tensile samples is shown in Figure 3b–f. All experimentally fractured SS316L lattice samples, regardless of lattice thickness, exhibit a clear approximately 45° fracture pattern (Figure 3f), indicative of shear-dominated failure under uniaxial tensile loading. This shear orientation is typical for ductile metallic materials, where plastic deformation localizes along maximum shear stress planes.

To validate and further understand this observation at the atomic scale, we selected the t = 0.5 mm lattice as a representative model in MD simulations. The evolution of shear strain during tensile loading is shown in Figure 3b–e, which maps the atomic shear strain fields at various global strain levels. Initially, the structure remains undistorted (0% strain), but by 8.4% global strain, local shear bands begin to emerge, particularly at lattice node intersections. As deformation progresses (10.0–14.7% strain), shear strain accumulates at a ~45° angle to the loading direction, aligning with the experimentally observed fracture planes. This shear band formation is typical in metallic lattices where yielding initiates at stress-concentrated strut junctions and propagates through diagonal planes of maximum shear stress.

Notably, maximum shear strain values concentrate along these diagonal regions, and fracture initiates exactly at these sites, consistent with experimental failure locations. This agreement between macro-scale fracture patterns and atomic-level shear localization provides strong validation of the ductile failure. It also confirms that the deformation and failure of bioinspired SS316L lattice structures are governed by geometric stress concentrations and shear-dominated mechanisms, irrespective of scale. These findings support the use of MD simulations not only for property prediction but also for visualizing early damage initiation, guiding future lattice architecture optimization for enhanced strength and damage tolerance. While direct comparisons are limited by the disparity between simulation and experimental scales, the parallel trends provide important validation of the structure–property relationships observed experimentally. Thus, MD simulations offer unique insight into underlying deformation mechanisms, informing design guidelines for tuning mechanical properties in additively manufactured metallic lattices.

### 3.5. Tensile Performance Comparison Between SS316L-Cu and SS316L Lattice Structures

**Figure 4:**
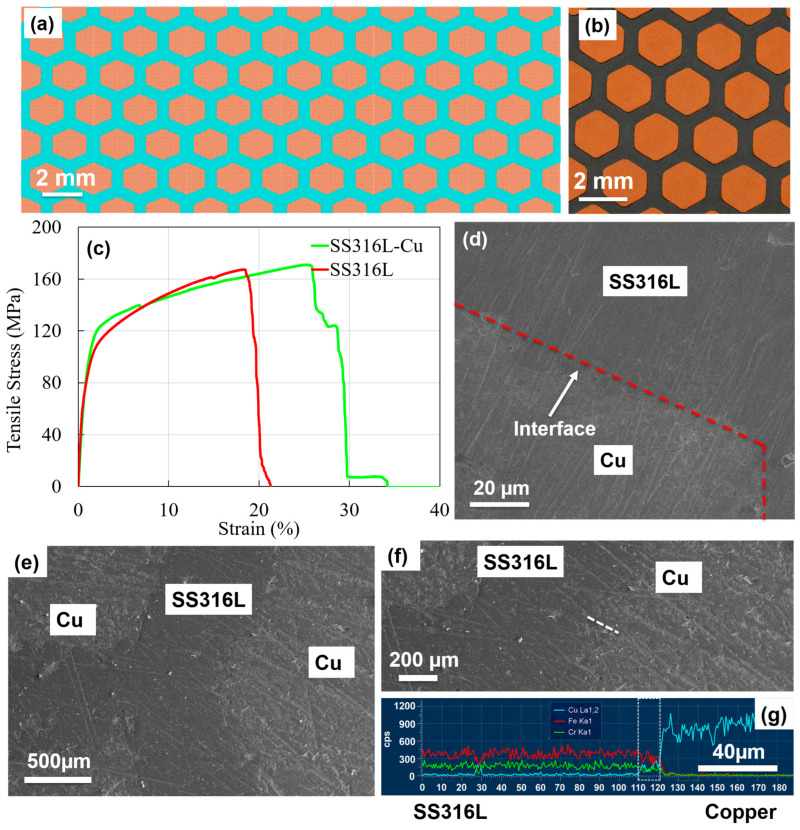
(**a**) Computer model of SS316L-Cu multimaterial, (**b**) manufactured SS316L-Cu multimaterial, and (**c**) comparison of tensile response of ss316L and SS316L-Cu multimaterials, (**d**) SEM micrograph showing interface (**e**,**f**) SEM micrograph used for EDS scan, and (**g**) EDS line scan at SS316L-Cu interface.

The tensile stress–strain behavior of SS316L lattice structures and SS316L-Cu multimaterial composites, both fabricated with a lattice wall thickness of 0.75 mm, which was selected as the reference thickness for comparative evaluation, is shown in Figure 4. It is worth noting that the coefficient of thermal expansion values of SS316L and Cu are relatively close, approximately 16.0 × 10^−6^ K^−1^ and 16.8 × 10^−6^ K^−1^, respectively [54,55]. This small difference indicates good thermal compatibility between the two materials and suggests that significant thermal mismatch stresses are not expected during cooling after Cu infiltration. Therefore, the SS316L-Cu multimaterial structure is expected to maintain favorable interfacial compatibility, supporting effective load transfer across the interface during mechanical deformation as observed in Figure 4.

The SS316L lattice structure (red line) exhibited an ultimate tensile strength (UTS) of approximately 166 MPa, while the SS316L-Cu multimaterial (green line) reached a peak of around 170 MPa. Although this increase in strength is modest, it suggests that copper infiltration preserves, and may slightly enhance, the load-bearing capability of the original SS316L lattice structure. The most significant distinction between the two systems lies in ductility, or strain at failure. The monolithic SS316L lattice failed at a strain of ~20%, whereas the SS316L-Cu composite endured deformation up to ~30%, marking a substantial ~50% improvement in ductility. This enhancement is attributed to the infiltrated copper phase, which offers increased cross-sectional area, helping to distribute stress more uniformly and delay the onset of fracture.

Scanning electron microscope (SEM) and energy-dispersive X-ray spectroscopy (EDS) line-scan analyses were performed to investigate Cu infiltration and elemental distribution across the SS316L–Cu interface. The EDS results showed Fe, Cr, and Cu compositional transitions across the interface region, indicating the formation of an interfacial diffusion/bonding zone of approximately 10 µm. The presence of this diffusion region suggests metallurgical interaction between SS316L and Cu, which may facilitate load transfer and influence deformation behavior within the multimaterial system. The improved tensile ductility and extended post-yield behavior observed in SS316L–Cu structures suggest that interfacial bonding contributes to stress redistribution and enhanced deformation accommodation. The localized deformation behavior within the SS316L–Cu multimaterial structure may also contribute to the observed tensile response. The infiltrated Cu phase may serve as a compliant region capable of redistributing localized stresses and reducing stress concentrations near lattice node intersections and strut junctions. Furthermore, the extended post-yield deformation observed in SS316L–Cu samples suggests progressive localized plastic deformation and gradual damage accumulation rather than catastrophic fracture. Additionally, MD simulations provide complementary atomistic insights into localized deformation mechanisms, including atomic rearrangement and local stress redistribution near material interfaces during deformation. Although these simulations do not directly represent macroscopic infiltration behavior, they help explain possible local deformation processes contributing to the overall tensile response. Such cooperative deformation behavior indicates improved damage tolerance in the SS316L–Cu multimaterial architecture. Future studies may consider investigating the effects of processing temperature and post-processing heat treatments on diffusion characteristics and their influence on the mechanical behavior of SS316L–Cu multimaterial systems.

Additionally, the SS316L-Cu sample demonstrated a more gradual and extended post-yield behavior, with observable stress drops and plateaus indicative of progressive microstructural damage and plastic deformation. In contrast, the SS316L lattice experienced a relatively sharp fracture following peak stress. These findings indicate the effectiveness of copper infiltration in enhancing the toughness of lattice-based SS316L structures without sacrificing strength. The resulting SS316L-Cu multimaterial holds significant promise for load-bearing applications where both structural integrity and damage tolerance are critical. The present study demonstrated a hybrid manufacturing approach combining LPBF and molten Cu infiltration to fabricate SS316L–Cu multimaterial structures and investigate their mechanical behavior. Bioinspired honeycomb structures were selected as a representative architecture as they provide a controllable framework for systematically evaluating the influence of geometry on mechanical performance and multimaterial interactions. While the current work focused on demonstrating the feasibility of the proposed multimaterial concept and its effect on tensile properties, future studies can further expand this approach by exploring additional architected geometries such as TPMS and other three-dimensional lattice structures. Furthermore, post-processing techniques including heat treatment and surface modification may provide additional opportunities for tailoring interfacial characteristics and overall mechanical performance. Such future developments may support the design of next-generation multifunctional metallic multimaterials for structural, thermal, and energy-related applications.

## 4. Conclusions

This study presents a comprehensive investigation into the design, fabrication, and mechanical behavior of bioinspired SS316L-Cu multimaterial structures produced through a hybrid additive manufacturing process. The key findings are summarized as follows:

1. Hybrid manufacturing via LPBF and copper infiltration was successfully implemented to fabricate bioinspired honeycomb structures, demonstrating feasibility for complex metallic multimaterials with enhanced properties.
2. Lattice wall thickness was found to be a critical design parameter, with tensile strength and Young’s modulus increasing significantly as wall thickness increased from 0.25 mm to 1.00 mm.
3. Copper infiltration improved ductility by approximately 50% compared to the monolithic SS316L lattices, without a substantial reduction in tensile strength, confirming its effectiveness in enhancing toughness.
4. Molecular dynamics simulations validated the experimental trends, revealing consistent increases in tensile strength with lattice thickness and offering atomistic insights into deformation and failure mechanisms.

These findings establish an understanding of structure–property relationships in SS316L-Cu multimaterial and offer design guidelines for next-generation multifunctional metallic components in structural and thermal applications.

## Data Availability

The original contributions presented in this study are included in the article/supplementary material. Further inquiries can be directed to the corresponding author.

## Supplementary Materials

The following supporting information can be downloaded at: https://www.mdpi.com/article/10.3390/mi17060699/s1, Figure S1: Melting furnace and Vacuum system to manufacture hybrid SS316L-Cu samples; Figure S2: (a) LPBF fabrication of duplicate SS316L lattice specimens under identical processing conditions (t = 0.5 mm), and (b) comparison of tensile stress–strain responses showing high repeatability between independent samples with mechanical property variation below 4%; Figure S3: Optical image of fractured SS316L–Cu multimaterial specimen; Section S1: Copper Infiltration process; Section S2: Repeatability Assessment; Section S3: Deformation Behavior of SS316L–Cu Multimaterial Structures.
