# Supplementary material for: Manufacturing of Bioinspired SS316L-Based Multimaterials: Processing, Mechanical Properties and Modeling
Source: Micromachines (Basel). 2026 Jun 8;17(6):699. doi: 10.3390/mi17060699 (PMC13304474; doi:10.3390/mi17060699)
Supplement: Supplementary file 1 [file micromachines-17-00699-s001.zip › micromachines-4298000-supplementary.pdf]

Manufacturing of Bioinspired SS316L-Based Multimaterials: Processing, Mechanical Properties and Modeling

Vinod Kumar Darapureddy [1], Tuhin Mukherjee [2] , Sonia Mary Chacko [3] and Zahabul Islam [1,*]

[1] Mechanical and Manufacturing Engineering, School of Engineering, Bowling Green State University, Bowling Green, OH 43403, USA
[2] Mechanical Engineering, Iowa State University, Ames, IA 50011, USA
[3] Robotics Engineering, School of Engineering, Bowling Green State University, Bowling Green, OH 43403, USA
[*] Correspondence: mdzisla@bgsu.edu

Supplementary

S1 Copper Infiltration process:

Following LPBF fabrication, the SS316L lattice structures were subjected to a Cu infiltration process to fabricate SS316L–Cu multimaterial structures. SS316L lattices were initially fabricated using manufacturer-recommended optimized LPBF process parameters, as described in Section 2.2, to ensure high-density fabrication quality. A mold-assisted infiltration approach was employed for Cu incorporation. High-purity Cu was melted at 1130°C and infiltrated into the lattice structure using gravity feeding together with vacuum assistance (approximately −30 psi) to improve liquid metal penetration throughout the porous architecture. Following infiltration, the samples were allowed to cool naturally to room temperature prior to mechanical testing and characterization.

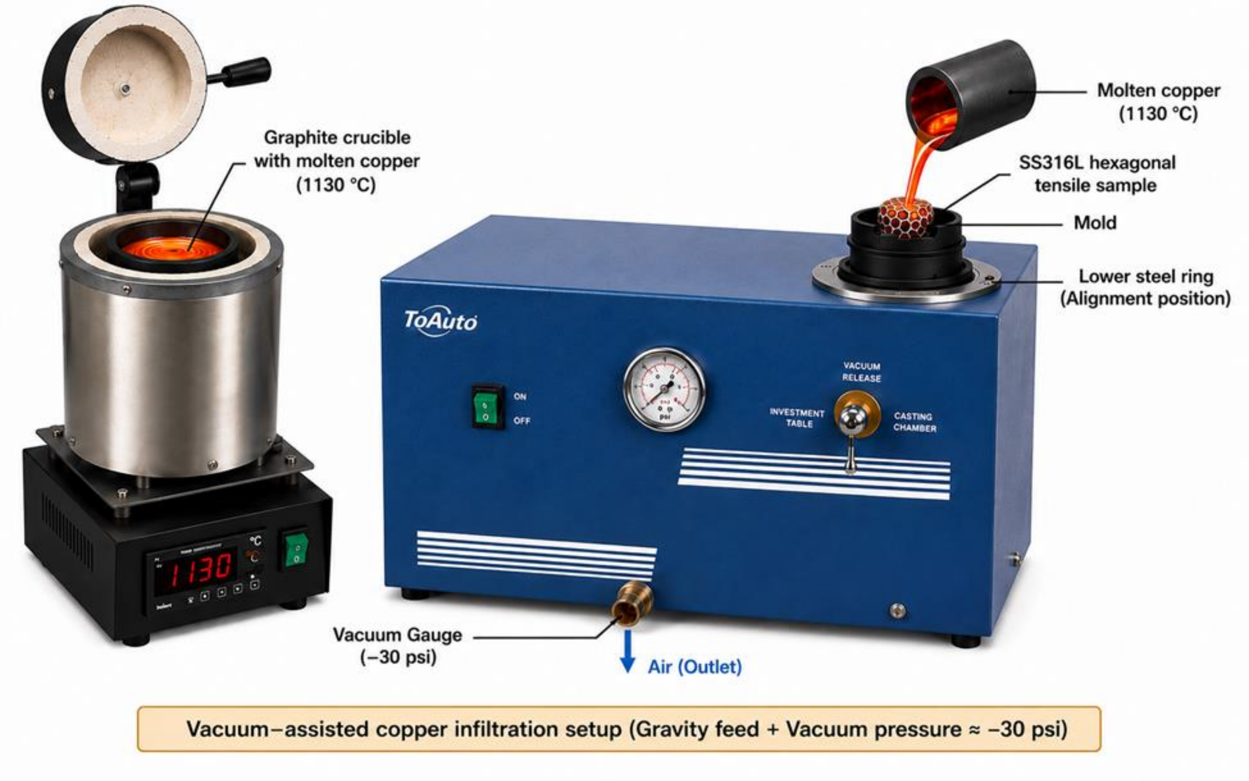

Figure S1. Melting furnace and Vacuum system to manufacture hybrid SS316L-Cu samples.

S2 Repeatability Assessment

To evaluate repeatability and mechanical reliability of the LPBF process, additional tensile experiments were performed on independently fabricated specimens under identical processing conditions as discussed in section 2.2. As a representative validation case, two SS316L lattice specimens with wall thickness (t = 0.5 mm) were manufactured and tested. The tensile responses demonstrated good agreement, with variations in tensile strength, Young's modulus, and yield strength remaining below 4%. These results indicate good repeatability of the LPBF fabrication process and mechanical testing procedure.

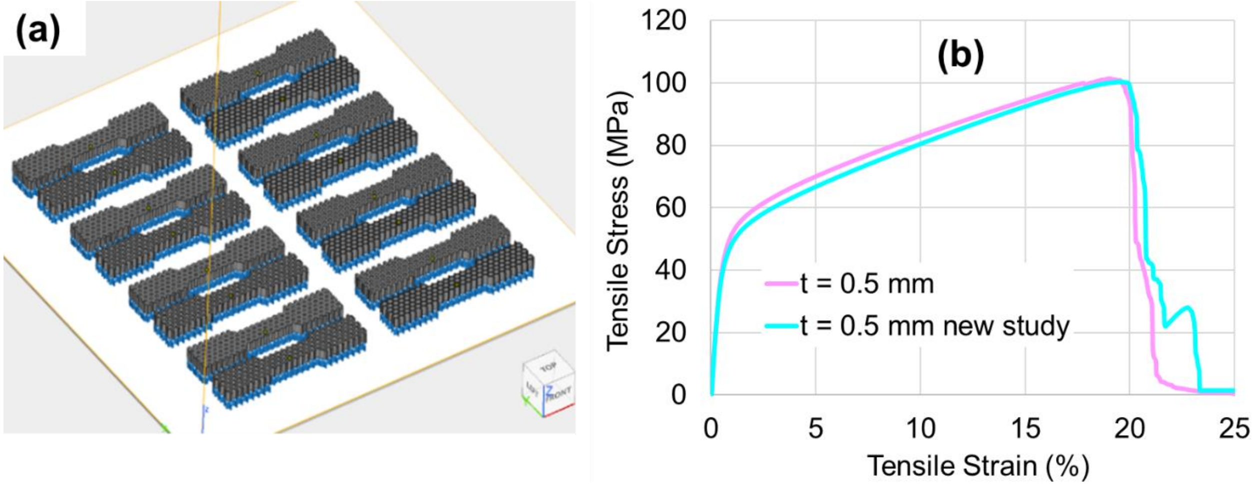

Figure S2: (a) LPBF fabrication of duplicate SS316L lattice specimens under identical processing conditions (t = 0.5 mm), and (b) comparison of tensile stress–strain responses showing high repeatability between independent samples with mechanical property variation below 4%.

S3. Deformation Behavior of SS316L–Cu Multimaterial Structures

To provide additional insight into the deformation behavior and possible load-sharing mechanisms during tensile loading, fractured SS316L–Cu multimaterial specimens were examined using optical observations. As shown in Figure S3, the region adjacent to the fracture surface shows honeycomb cell fracture along the loading direction. This deformation behavior suggests that the SS316L lattice experienced progressive plastic deformation prior to final fracture rather than abrupt catastrophic failure. To provide a simplified interpretation of the load-sharing behavior, a rule-of-mixtures-based representative volume element (RVE) approach was considered. For the lattice structure with wall thickness t = 0.75 mm, the volume fraction of SS316L was

approximately 0.6286, while the Cu phase occupied the remaining volume fraction of approximately 0.3714. Using the experimentally measured tensile strength of the SS316L lattice (~166 MPa) and reported Cu tensile strength values (~160–200 MPa), the estimated composite strength based on the rule of mixtures can be expressed as:

$$\sigma_{SS316L-Cu} = \sigma_{SS316L,lattice} V_{f,SS316L,lattice} + \sigma_{Cu} V_{f,Cu} \quad \text{(S1)}$$

The estimated composite strength ranged from approximately 164–179 MPa, which agrees reasonably well with the experimentally observed tensile strength of approximately 170 MPa for the SS316L–Cu multimaterial structure. This agreement supports the interpretation that the infiltrated Cu phase participates in load sharing together with the SS316L lattice framework. Initially, the SS316L lattice likely acts as the primary load-bearing structure because of its relatively higher stiffness, whereas the infiltrated Cu phase progressively contributes to stress redistribution and deformation at larger strains. Owing to its relatively high ductility and ability to sustain large plastic strains, the Cu phase may continue carrying load even after localized plastic deformation develops within the lattice structure. Such cooperative deformation behavior may contribute to the extended post-yield response and enhanced ductility observed in the SS316L–Cu multimaterial system.

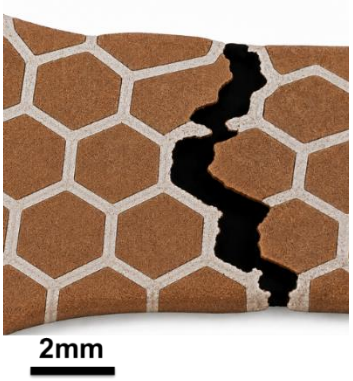

Figure S3. Optical image of fractured SS316L–Cu multimaterial specimen.
